# New Ways for Patients to Make Sense of Their Electronic Health Record Data Using the Discovery Web Application: Think-Aloud Evaluation Study

**DOI:** 10.2196/41346

**Published:** 2023-04-03

**Authors:** Drashko Nakikj, David Kreda, Nils Gehlenborg

**Affiliations:** 1 Department of Biomedical Informatics Harvard Medical School Boston, MA United States

**Keywords:** patient, sensemaking, electronic health records, personal health records, multiple providers, design

## Abstract

**Background:**

In the United States, patients can access their electronic health record (EHR) data through patient portals. However, current patient portals are mainly focused on a single provider, with very limited data sharing capabilities and put low emphasis on independent sensemaking of the EHR data. This makes it very challenging for patients to switch between different portals and aggregate the data to obtain a complete picture of their medical history and to make sense of it. Owing to this fragmentation, patients are exposed to numerous inconveniences such as medical errors, repeated tests, and limited self-advocacy.

**Objective:**

To overcome the limitations of EHR patient portals, we designed and developed Discovery—a web-based application that aggregates EHR data from multiple providers and present them to the patient for efficient exploration and sensemaking. To learn how well Discovery meets the patients’ sensemaking needs and what features should such applications include, we conducted an evaluation study.

**Methods:**

We conducted a remote study with 14 participants. In a 60-minute session and relying on the think-aloud protocol, participants were asked to complete a variety of sensemaking tasks and provide feedback upon completion. The audio materials were transcribed for analysis and the video recordings of the users’ interactions with Discovery were annotated to provide additional context. These combined textual data were thematically analyzed to surface themes that reflect how participants used Discovery’s features, what sensemaking of their EHR data really entails, and what features are desirable to support that process better.

**Results:**

We found that Discovery provided much needed features and could be used in a variety of everyday scenarios, especially for preparing and during clinical visits and also for raising awareness, reflection, and planning. According to the study participants, Discovery provided a robust set of features for supporting independent exploration and sensemaking of their EHR data: summary and quick overview of the data, finding prevalence, periodicity, co-occurrence, and pre-post of medical events, as well as comparing medical record types and subtypes across providers. In addition, we extracted important design implications from the user feedback on data exploration with multiple views and nonstandard user interface elements.

**Conclusions:**

Patient-centered sensemaking tools should have a core set of features that can be learned quickly and support common use cases for a variety of users. The patients should be able to detect time-oriented patterns of medical events and get enough context and explanation on demand in a single exploration view that feels warm and familiar and relies on patient-friendly language. However, this view should have enough plasticity to adjust to the patient’s information needs as the sensemaking unfolds. Future designs should include the physicians in the patient’s sensemaking process and improve the communication in clinical visits and via messaging.

## Introduction

### Background

Typically, people in the United States encounter multiple providers during the course of their lives [1]. Although for most of their lives they likely interact with several providers, as they become older this number can rapidly increase and reach tens of providers [2]. Consequently, a patient’s electronic health record (EHR) data are often distributed among many providers that have none or poor data sharing among them [1].

This fragmentation causes multiple hurdles for the patients and clinicians to reliably access and meaningfully use EHR data in safe ways. Patient portals through which patients access the EHR data from their providers are empowering tools [3,4]; however, they are traditionally tied to a single provider [5]. This makes it extremely challenging for the patients to aggregate the EHR data for obtaining a complete picture of their medical history and to make sense of it [6]. Patients are forced to hop between different portals to put the pieces together for a holistic view of the data related to their medical issues through a tedious and frustrating process that often gets aborted [7,8]. Clinicians face a similar challenge; although they can access EHR data within the walls of the institution they work for, going outside those boundaries for accessing patient data might be very difficult [9]. Such fragmentation of the patients’ EHR data is setting up a stage for multiple problems that pose burden and threats to the patients, providers, and the health care system overall [8]. These include threats to the patients such as errors owing to the lack of awareness about their medical history across different providers, additional costs such as repeated or redundant tests because of missing information, and limited advocacy owing to lack of understanding and ownership of their own data [1,6,8,10].

Some of these problems might be mitigated or avoided if the patients have access to their data at all times, without any restrictions. Fortunately, the United States government recognized the benefits patients’ ownership of their EHR data might bring and passed laws that will require all providers to enable EHR data access by the end of 2022 [11,12]. Through their patient portals credentials and using third party apps, the patients will be able to access their EHR data, which includes laboratory results, vital signs, conditions, procedures, medications, and many others, from any provider [13]. Although this may sound very empowering, there is still an open question about how this information will be returned to the patients to be used in a meaningful way.

Patient portals have been valuable to the patients, but are typically linked to a single provider and hence have a limited capacity to offer patients sensemaking of their complete EHR data. Literature has shown that patient portals such as MyChart (from Epic), My HealtheVet (from the Veterans Health Administration), and My Health (from Vanderbilt University Medical Center) brought benefits in the domains of consumer empowerment [4], patient engagement [14], and health care communication [3], and delivered a sense of trust in and improved collaboration with providers [5]. Patient portals have been able to enhance understanding of medical conditions, simplify and clarify patients’ instructions, and provide a greater control over health outcomes, among others [3]. Similarly, they have played a key role in improving patient-provider communication and timely information sharing for clinical decisions [3]. Existing patient portals achieve the previous benefits by appointment scheduling, enabling message-based communication with the providers, and collecting various medical data and making it available to the patients. Contemporary patient portals have made major strides in securing access to vital signs, laboratory results and other diagnostics tests, medication lists, and radiology images and reports, among many other data types [15,16]. These data are typically presented with basic, noninteractive visualizations to support finding insights form the data or used to generate alerts that raise the awareness of the patients [6,17]. In addition, with the increasing popularity of Open Notes [18], patients are now able to see their clinical notes and visit summaries for increased participation in shared decision-making [19].

However, these portals were not designed to work on EHR data from multiple providers. Although their features can be very useful, they are not addressing important aspects of sensemaking for EHR data from multiple providers, such as filtering by and comparison across providers or providing powerful visual analytics mechanisms to support the dissection of and navigation through these complex data [6]. In addition, researchers also identified a number of improvements that are necessary for higher adoptions of patient portals and related to making sense of the EHR data, such as more context while exploring the data, substantially improved usability, and deployment of language that is easy to understand [6]. Although existing studies take a broad approach and focus mostly on learning about feature use in patient portals [20], barriers to adoption [21], and desired features [6,21], it appears that we have neglected to deeply understand how patients actually go about making sense of their EHR data, especially from multiple providers, and find solutions that address this need at a very coarse level.

All this necessitates a strong push toward deep understanding of the patients’ sensemaking needs, as well as designing and developing patient-facing applications that access and present their EHR data from multiple providers. These applications should make the data available in a way that enables the patients to easily make sense of it, plan ahead, and take well-informed actions through a shared decision-making process with their physicians. Recently, third party patient-facing applications for making sense of EHR data from multiple providers have been emerging. These applications were designed for smartphones, such as Apple Health Records [22], iBlueButton [23], and OneRecord [24], as well as for desktop and laptops, such as 1upHealth [25]. However, these applications generally do not make strong connections between the medical records and the time they were recorded. Although they do cover the longitudinal aspect of these data by plotting time series of vital signs and laboratory results [22,24] and provide insights in their periodicity, they lack the capability for detecting co-occurrence of medical events and pre-post analysis. These apps most often provide easy recognition and explanations of abnormal values [24,25], but are less capable of providing deeper context of individual medical records and temporal or semantic relationships between them. Similarly, although they provide capabilities for looking at the data from different providers [22-24], it remains challenging to make easy comparisons across providers and multiple record types.

### Objectives

**Figure 1:**
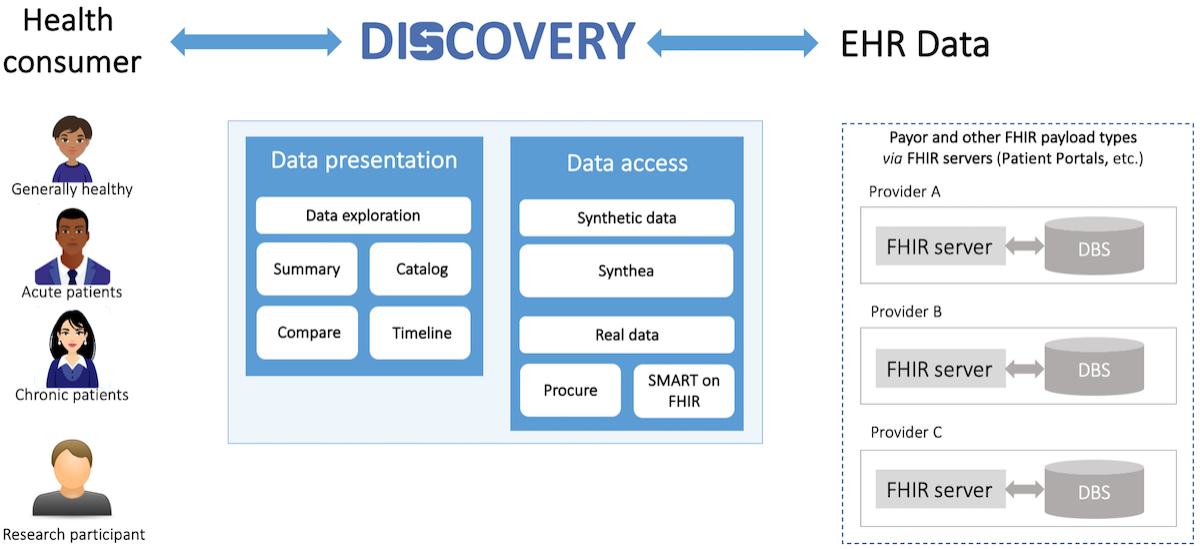
Discovery’s users and architecture. Discovery targets everyday health consumers and research participants. The data access layer can pull data from Fast Health Care Interoperability Resources (FHIR) servers and feed the presentation layer, which displays that data using multiple views: Summary, Catalog, Compare, and Timeline. DBS: data base system; EHR: electronic health record; SMART: Substitutable Medical Applications and Reusable Technologies.

To overcome these limitations, and potentially improve sense of ownership and control of patients’ own EHR data, increase their health awareness, and heighten proactivity and empowerment for clinical visits, we designed and implemented an open-source web application for desktops and laptops, called Discovery [26] (Figure 1). Discovery targets a wide user base, including the everyday health consumers that can be generally healthy individuals, acute and chronic patients, as well as research study participants. Discovery is able to pull the patient’s EHR data from different providers and enable the patient to independently, that is, without a clinician or other expert present, explore, and make sense of their data. To enable such capabilities, Discovery relies on 4 views (user interface [UI] layouts) for looking at the data: Summary, Catalog, Compare, and Timeline; each with their own strengths for helping the user to answer different types of questions.

To evaluate the extent to which Discovery is able to meet the patients’ needs and learn more about how to support sensemaking of EHR data from multiple providers, we asked the following research questions (RQs):

1. *How well is Discovery supporting the sensemaking of EHR data:* Is the application helpful for patients to make sense of their medical records from multiple providers? (RQ1)
2. *What tasks do patients want to successfully complete when making sense of their EHR data:* What are the functionalities they need from a sensemaking support application like Discovery? (RQ2)
3. *What interaction mechanisms are suitable for the patients:* What should the user interface look like and what component should it have? (RQ3)

To answer these RQs, we conducted a study with 14 participants. Each of the participants went through a 60-minute evaluation session of Discovery. On the basis of the think-aloud protocol [27], the participants were asked to complete a variety of sensemaking tasks using Discovery for a fictitious patient with synthetic data [28]. After this block, the participants provided semistructured feedback on their experience using Discovery and possibilities for improvement. The transcribed audio materials, the annotated video recordings of the participants’ interactions with Discovery, and the in-session notes were combined and thematically analyzed.

### Principal Findings, Design Implications, and Contributions

We found that Discovery provided much needed features for EHR data sensemaking support and could be used in a variety of everyday scenarios, especially when preparing for and in clinical visits and also for raising awareness, reflection, and planning. According to the participants, Discovery provided a robust set of features for supporting independent exploration and sensemaking of their EHR data: summary and quick overview of the data, finding prevalence, periodicity, co-occurrence, and pre-post of medical events, as well as comparing medical record types and subtypes across providers, anchored to a timeline. However, the interface appeared clinical, somewhat complicated and unintuitive at times, with a noticeable learning curve for most of the participants. This was primarily because of the multiple views for data exploration that, although very useful independently, had some overlaps in functionality and caused difficulties in determining which one to use for the questions at hand. Another factor was Discovery’s well implemented, but highly customized UI controls, and powerful widgets that look the same, but operate slightly differently across different views.

This study resulted in a set of design implications toward improving the user experience for Discovery; making it more intimate through personalization and familiar UI controls and interactions, consolidating the multiple views into one that is centered on the Catalog view while preserving the functionalities of the rest, and adding more context while browsing.

The findings and the design implications from this study are applicable not only to Discovery but also to any new efforts for building patient-facing tools and applications that support making sense of EHR data from multiple providers. This work provides the following contributions to the fields of biomedical informatics and human-computer interaction: (1) *novel patient-facing application for supporting sensemaking of EHR data from multiple providers;* (2) *design guidelines, interaction principles, and preferred features for successfully accomplishing* (1)*, and* (3) *broader idea of what making sense of the EHR data from multiple providers entails for patients and what the ultimate purpose of such endeavor is.*

## Methods

### Description of Discovery

Discovery is an open-source web-based application that is suitable for desktops and laptops. It is a product from the *All of Us* project’s [29] desire to return EHR information to their research participants under the auspices of the *Sync for Science* initiative [30].

Discovery is able to pull the patient’s EHR data from different providers in a single point of access and enable the patient to independently explore and make sense of and understand their data. In its design, Discovery relies on well-established framework about how analysts perform sensemaking of information [31]. Sensemaking is a cognitive process that involves continuous exploration of complex artifacts to surface meaning that produces concrete actions. On the basis of the sensemaking framework, the analysts engage in 2 main activities: foraging loop and sensemaking loop. In the foraging loop, the analyst is trying to identify relevant pieces of information for the open question. In the sensemaking loop, the analyst is trying to use that relevant information and establish connections that will help them find an answer to the question. In case of insufficient evidence, the analyst engages in another round of foraging, and the cycle repeats. With respect to this framework, the key aspect of the sensemaking process that Discovery supports is efficient foraging—finding the relevant pieces of information for a given question. It is left to the user to further organize that information in a meaningful way for future use.

Existing literature and applications were our main sources for the design of Discovery. We observed that the features in existing patient portals are not sufficient for independent sensemaking of the EHR data. In addition to being fed with simple data patterns detected and presented by the application, patients should also be able to look for patterns by themselves [6,32]. Consequently, and keeping in mind that Discovery should support enormous variability of information needs [6,10], we decided to stratify those and provide separate data views (UI layouts) to address them. As a part of our design process, we had a 1-hour brainstorming session with 9 participants that were asked to think of all possible questions they would ask if they had all their EHR data at their fingertips. The session produced >100 questions that were thematically analyzed and classified. This resulted in several archetypes of questions that deserved their own Discovery views to be answered efficiently. In the design of these specialized views, we borrowed and simplified ideas from existing work on visualizing EHR data for clinicians [33,34] and personal health data [35] and adjusted them to the everyday consumer based on points raised in previous literature reviews [36,37].

#### Data Access Layer

Discovery is built to work on data that follow the Fast Health Care Interoperability Resources (FHIR) standard [38]. Currently, Discovery is designed to work with a limited set of structured data from the United States Core Data for Interoperability Standard [13], disregarding clinical notes for the time being. Discovery can access synthetic patient EHR data using Synthea [39], and real EHR data through Procure [40] or directly through the Substitutable Medical Applications and Reusable Technologies (SMART) on FHIR protocol [38]. For the purposes of our evaluation study, we used the access to synthetic EHR data option. To achieve this, we built FHIR servers that generate fake data for fictitious patients that Discovery can access and pull data from.

The data aggregation in Discovery takes a very simple form where accounting for identification of missing, conflicting, or redundant information is not taken into consideration. After Discovery pulls the EHR data from multiple providers, it basically has a “bag” of FHIR resources that we call records.

#### Data Presentation Layer

The presentation layer on the client side is built in React. To achieve efficient data exploration, Discovery relies on 4 different views of the patient’s EHR data: Summary, Catalog, Compare, and Timeline. Each of these views has their own unique strengths and provides better support than the others for answering particular types of questions.

### Summary View

**Figure 2:**
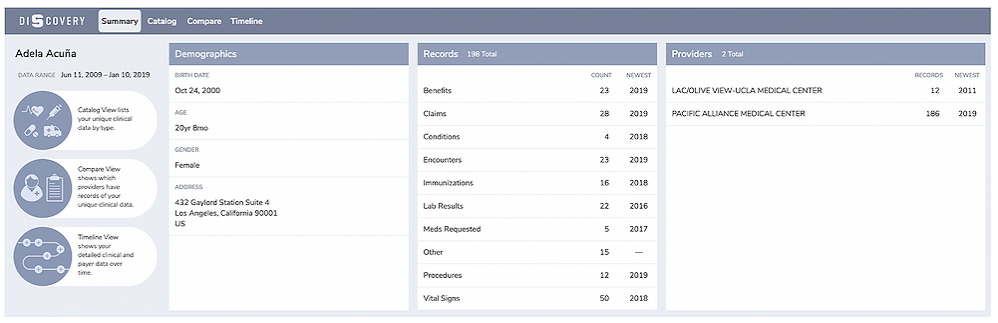
The Summary view for the fictitious patient Adela Acuna and the high-level overview of her synthetic data.

The Summary view provides a high-level overview of the patient’s medical records (Figure 2). These data are displayed in 3 data categories: Demographics, Records, and Providers. The Demographics category provides the personal information about the patient, such as date of birth, age, gender, and address. The Records category shows the different record types the patient has in their data, listed in alphabetical order. In this, we show the total number of records available and the number of records by type. Next to each record type count, we also provide the year when the last record for that type was created. The Providers category shows the data from different providers and the total number of records with each of them. Similar to the Records category, we show the year when the last record for a given provider was created.

To the left of the data categories, there is a list of the other 3 views available in Discovery: Catalog, Compare, and Timeline, with a short description for what each of them does.

The types of questions that the Summary view is designed to answer efficiently are the following:

1. What does my data look like on a high level—how many records and what types, from which providers, and for what period?
2. What is the latest data that I have?
3. Is my demographics information correct and up to date?

### Catalog View

**Figure 3:**
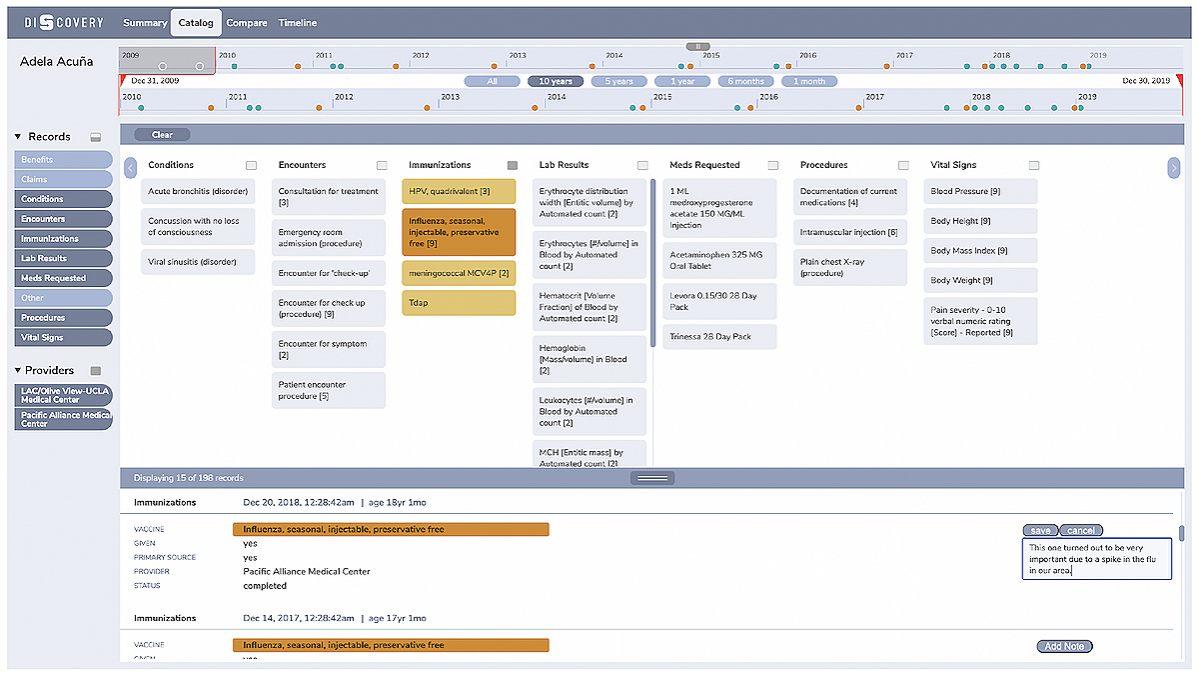
The Catalog view with filters on the left and the timeline widget on the top. In the middle, there is a grid-like presentation of the records defined by the filters and the timeline organized in columns by record type. The user selected all immunizations to inspect in more detail in the Details Panel (yellow), the Influenza being the last selection (orange). This selection is also reflected in the timeline, with the same color coding of the dots that represent the days the immunizations took place.

Catalog view provides a catalog of the available records for efficient exploration (Figure 3). It has a Details Panel to show the records of interest in more detail and a timeline to indicate when those events took place.

#### Scoping the Records

The user scopes what records to look at with the filters on the left and the timeline widget on the top (Figure 3). By default, all filters are turned on and the timeline is set to the full available time span, which lets the user see all the records they have in their data. However, by turning some of the filters off and changing the window in the timeline, the user can limit what records are displayed. Note that the user can also specify the provider or providers they want to see records from. In the timeline, they can specify a window width, that is, 1 month, 6 months, 1 year, 5 years, 10 years, or all, and move that window to a specified location in the timeline. If the window is different from “all,” then there is a secondary, zoomed-in timeline, right below the main one.

In the case from Figure 3, the user decided not to look at benefits, claims, and other, and wanted to see just the last 10 years of their data.

#### Data Organization in the Catalog

At the highest level, we have the record types that are laid out as columns in the view—conditions, encounters, immunizations, etc (Figure 3). In each column, we have Cards with labels that represent record subtypes—immunizations: human papillomavirus, quadrivalent; Influenza seasonal, injectable, and preservative free; meningococcal MCV4P; and Tdap. Each of those Cards can have ≥1 individual records, which belong to that same record subtype, but were created at different points in time—human papillomavirus, quadrivalent has 3 such instances.

#### Details Panel

**Figure 4:**
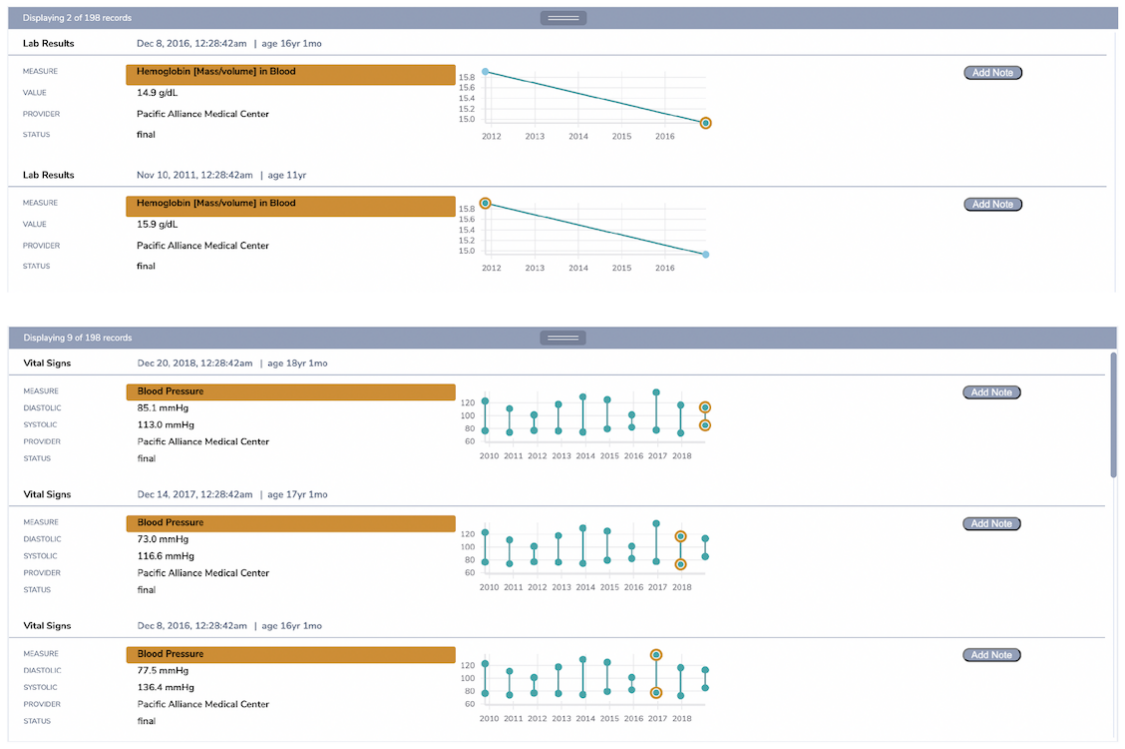
Top—laboratory results for hemoglobin with 2 instances and bottom—vital signs for blood pressure (diastolic and systolic) with 10 instances; plotted over time to give additional context for the individual records.

To inspect more details about a Card, the user can click on it and its individual records will show in the Details Panel, at the bottom of the view. In this panel, those records are displayed with basic information that provides the necessary context for making sense of them. It is worth noting that the FHIR resources have additional detailed information that we are not showing in this version of Discovery. When there is a longitudinal aspect of the records in a Card, in the case of laboratory results or vital signs, we provide a visualization of the values over time (Figure 4).

In addition, the user can put notes for the individual records to provide some additional context that was not captured by the EHR system of their provider.

Because a Card can have multiple records, and the user can select more than one Card at a time, the content in the Details Panel can quickly grow. To enable more efficient browsing within the Details Panel, the user can resize it vertically.

#### Synchronization Between the Details Panel and the Timeline

The records in the Details Panel are synced with the timeline such that they stand out by being colored in yellow. Note that the user can select multiple Cards at a time, but the records from the last selected one will be colored in orange to remind the user of their last interaction in the sensemaking process.

The types of questions that the Catalog view is designed to answer efficiently are the following:

1. What does my data look like at a more granular level?
2. Did some event happen to me (condition diagnosed, medication prescribed, or a procedure conducted, among others)? How many times and when?
3. Are there any patterns when those events took place (long time ago vs recently, every year, before or after some other event)?

### Compare View

**Figure 5:**
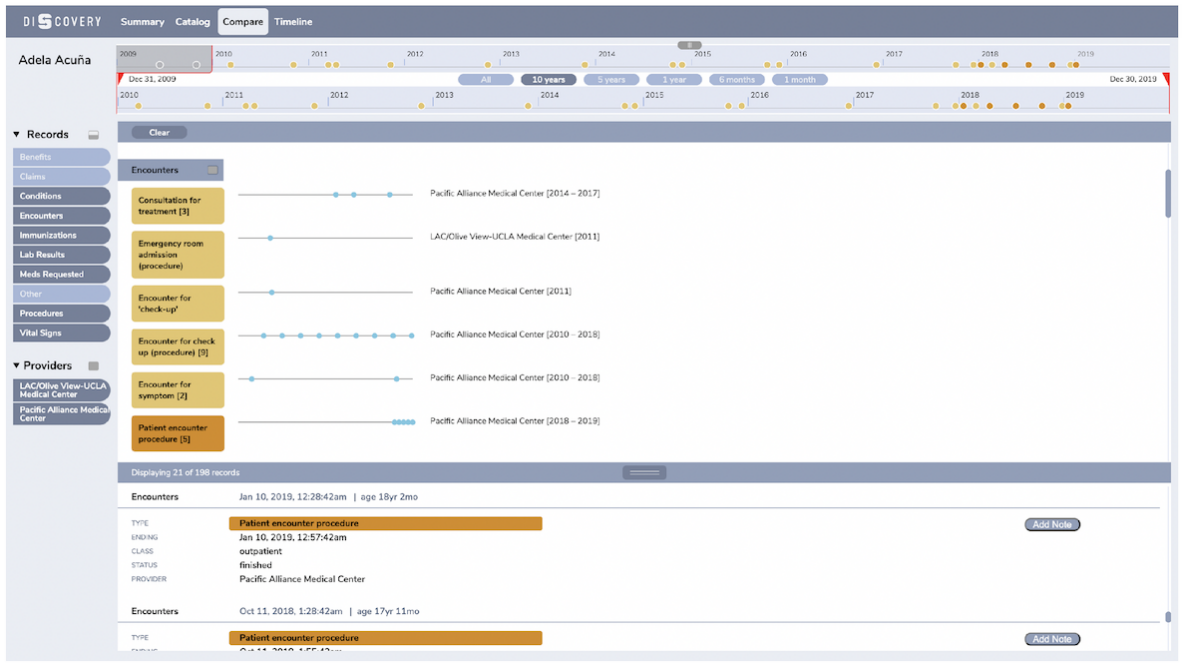
The Compare view where the user is comparing the different encounters across their 2 providers based on the sparklines to the right of the record Cards. Similar to the Catalog view (Figure 3), they selected all encounters for even more detailed comparison in the Details Panel.

Although the Catalog view is designed to be very powerful for efficient browsing through the data, it is less capable of comparing how that data looks across providers. To enable this functionality, we created a separate view called Compare view (Figure 5). Here, we use the same data organization as in the Catalog view, but instead of listing the record types as columns, we stack them on top of each other. We also add sparklines for each of the providers to the right of the record types to show when the data from a particular provider was created and for an easy comparison across providers. In case there is no data available from a given provider, there is no sparkline, but the ordering of the providers is still preserved. In the same manner, just like in the Catalog view, the user can interact with the Cards and see them displayed in more detail in the Details Panel, and reflected in the timeline.

The types of questions that the Compare view is designed to answer efficiently are the following:

1. How much data do I have compared across different providers (by record type)?
2. Which provider knows what about me?
3. Did I visit more than one provider for a given issue and when?

### Timeline View

**Figure 6:**
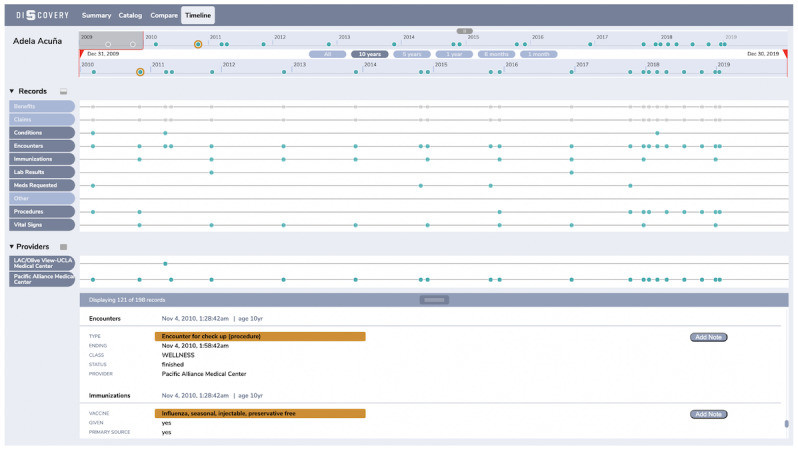
The Timeline view where the user is able to detect co-occurrence of events on the same day following the vertical alignment of the dots. Upon selection of a particular day in the zoomed-in timeline, the Details Panel is automatically scrolled to show the events that took place then.

Although the Catalog and the Compare view provide some insight when the medical events took place, it is still difficult to see what events happened on the same day and how are different medical events laid out and sequenced across records types. This is the limitation that the Timeline view is primarily designed to address (Figure 6).

In addition to the main timeline on the top, each of the selected filters has an independent timeline associated with them. This enables one to see a cumulative grid-like view about when medical events took place, what type of medical events were those, and if they co-occurred. This is achieved by vertically aligning the events that took place on the same day. The alignment starts from a dot for a specific date in the zoomed-in timeline and stretches downward across the timelines for the individual record types.

Although the Details Panel is still present here, it has a different role compared with the Catalog and Compare views. In the Timeline view, the Details Panel is prepopulated with the records specified by the filters and the time-window in the timeline and sorted by date in descending order. Similarly, the timeline also has a different role—rather than being static and showing when particular events took place, it is interactive and serves as a data browsing driver. The user can select a particular date by clicking on a dot in the timeline and the Details Panel will automatically scroll to that date and have the corresponding records shown in a sequential order.

The types of questions that the Timeline view is designed to answer efficiently are the following:

1. When was I mostly having medical events and of what type?
2. What events happened on the same day? What events happened before or after a specific event?
3. Did certain events happen on the same day or within a given time-window? What is the sequence of those events?

### Study Design

#### Ethics Approval

We obtained approval from our Harvard Faculty of Medicine institutional review board office to conduct this study (protocol number IRB20-1757). Each of the participants signed a consent form to participate in the study and was compensated with a US $20 Amazon gift card. The audio-video recordings of the interviews and the deidentified transcripts are kept on an encrypted and password protected machine. No one except the members of the research team have access to the data.

#### Participants

We recruited 14 study participants through advertisements on Craigslist. We chose this platform because it targets wide population and is not affiliated with any hospital. This was preferred to relying on recruiting patients through hospitals we have relationships with as it may bias the participants to sound more agreeable—if they feel a strong commitment to the care they receive from the hospital, or negative—if they recently had a bad experience. Moreover, we needed direct and flexible access to large pool of potential participants because we wanted to balance the sample and Craigslist has shown to be very practical for this in our previous work. Relying on our screening questions, we included relatively healthy individuals and patients with acute and chronic illness who belong to various age groups. On the basis of the research on insights gaining from usability testing [41] and similar research from the health care domain [42], we recruited 15 participants to ensure coverage of usability issues and richness of the findings. One of the participants did not show up for the study, thus resulting in a total of 14 participants. However, this number showed to be sufficient as we reached saturation in the data analysis.

The recruitment took place over the course of 3 weeks. The participants had to meet the following eligibility criteria: an adult fluent in English with a working laptop or desktop computer (with screen size ≥13”), a stable internet connection, normal vision or well-corrected vision with glasses or lenses, no color blindness, and medical records with one or multiple providers or institutions (hospitals or private clinical practices).

#### Procedures

**Figure 7:**
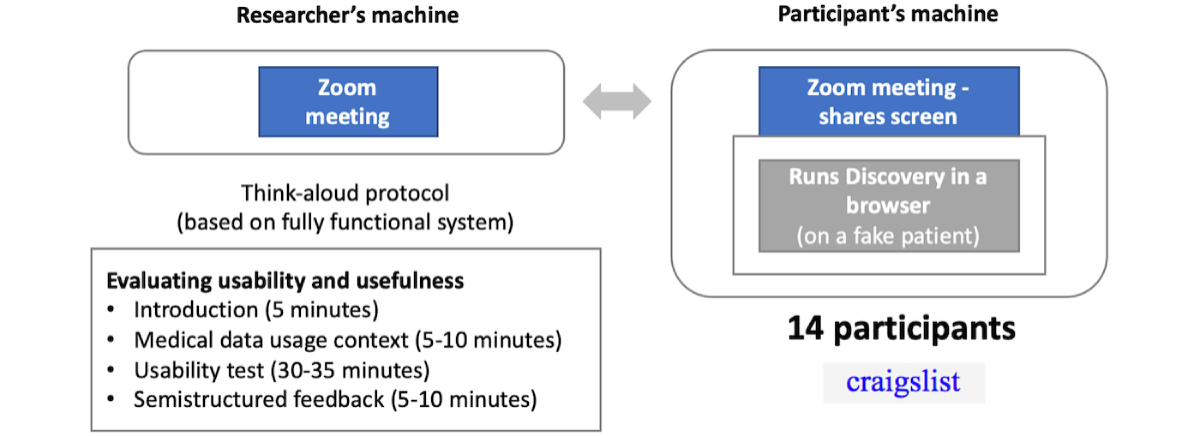
Study design.

We took inspiration from existing patient portal usability studies and tailored one that best suited our RQs [43-45]. Each of the participants went through a 60-minute evaluation of Discovery in a Zoom (Zoom Video Communications) meeting (Figure 7). With the permission of the participant, the researcher recorded the meeting. The researcher started with a 5-minute introduction of the study where they explained to the participant what to expect in the session. Then, the researcher spent the next 5-10 minutes in collecting basic demographic information and information that describes the participant as a digital health consumer (Section A; Textbox 1).

Upon collecting these data, in the next 30 minutes, the researcher moved to testing the usability of Discovery using the think-aloud protocol [27]. For this purpose, and to gain better insight in the intuitiveness and the learning curve of Discovery, the researcher only provided a high-level overview of the application and what its main goals were. They presented the participant with a fake patient with synthetic data, Adela Acuna, used for evaluating Discovery. The participants were asked to pretend they are Adela to elevate empathy and motivate them to exercise more effort in completing the sensemaking tasks. The participant was asked to open Discovery in their internet browser and share their screen with the researcher for observing and recording the interactions. There were 3 blocks of sensemaking tasks the participant was asked to complete (Section B; Textbox 1). The first block involved very basic sensemaking tasks mainly designed to familiarize the user with the data they will work with and the functioning of Discovery based on multiple views. The second block involved a little more complex tasks that involved looking up information, finding prevalence and frequency of medical events, as well as accessing more details for the individual records, thus required learning how the interface operates at a more granular level. The third and final block had the most complex sensemaking tasks such as comparison across providers, identifying co-occurring events, and pre-post analysis, hence requiring multiple, less obvious, interactions with the interface to complete them. These sensemaking tasks’ purpose was not to quantitatively measure the answers’ accuracy and time to complete, or any other form of performance. They were rather there to let the participants engage with Discovery for the first time without knowing much about it and enable us to obtain qualitative insights in its intuitiveness, learning curve, how the participants conceptualize a mental model of their EHR data, and strategize how to make sense of those data using the available features.

After completing the tasks and in the last 10 minutes of the study session, the researcher collected semistructured feedback about the participants’ experience using Discovery and suggestions for improvement.

**(A) The questions for understanding the user as a digital health consumer and (B) the sensemaking questions for evaluating Discovery in a think-aloud protocol.**

- Digital health consumer characterization
  1. What is your age?
  2. How would you describe your medical history—have you been seeing doctors a lot or not?
    - Do you have any chronic conditions—anything that makes you monitor your health more closely and have more frequent doctor’s visits over longer period?
  3. How many different providers or institutions have medical information about you?
  4. How hard would you say it is to keep track of your medical information from those providers or institution?
    - What would be the biggest barrier for doing that?
  5. How comfortable are you with technology?
    - Do you currently use any devices to keep track and make sense of your health or medical information?
    - What do you like and dislike about them?
- Sensemaking tasks for evaluating Discovery
  - Basic questions (overview of data, functioning of Discovery; 5 minutes):
    1. For what time span is there data available for you (Adela)?
    2. From how many providers is there data about you? What are the provider names?
    3. What is the total number of records available for this patient?
    4. How many different views are there to look at the data? What does each do?
  - Focused questions (lookup, prevalence, frequency, details; 10 minutes):
    1. How many times did you have influenza shot before? Did you have it last year? Did you ever have it more than once in a single year?
    2. How many different immunizations did you have? What about in the last 5 years? First 5 years?
    3. How many of the different immunizations came from the University of California, Los Angeles Medical Center?
    4. How many times did you have *human papillomavirus (HPV), quadrivalent* immunization?
    5. What is the status of each *HPV, quadrivalent* immunization?
    6. When was the first time you had a *meningococcal MCV4P* immunization?
  - Complex questions (comparison across providers, co-occurrence, pre-post; 15 minutes):
    1. Can you compare the number of encounters you had with both of your providers?
    2. Were there years when you encountered any provider very frequently? What period was that?
    3. When did most of your procedures take place?
    4. Were there any medications requested when you were diagnosed with *acute bronchitis (disorder)*? What about requests within a year of that event?
  - Usefulness and usability feedback (10 minutes):
    1. What capabilities of Discovery did you like? What was easy to do?
    2. What capabilities did you dislike? What was hard to do?
    3. How do you feel the multiple views affected your ability to answer the questions?
    4. Imagine you are a user of Discovery. What would you find it most useful for in a real-life setting? Can you give me concrete examples?
    5. What are some questions that you struggled answering using Discovery?
    6. Are there questions that come to mind you wish Discovery can support better?

#### Data Collection

We recorded the Zoom meetings to obtain audio recordings of the entire conversation and video recordings of the interactions with Discovery. The researcher also took notes during the meetings.

### Data Analysis

The audio-recorded sessions were transcribed using a professional service [46]. We also analyzed the video recordings to provide further context and deeper understanding of the participants’ comments during the think-aloud protocol. Similar to the study by Segall [44], these video annotations were added to the transcripts and the notes for thematic analysis [47]. The analysis involved starting by open-coding the textual data by the first author (DN) of the paper. The emerging categories were discussed and reconciled in a meeting with the second (DK) and the last author (NG) to surface and reflect the most important points in the perceptions and the use of Discovery’s features, as well as the way participants wanted to make sense of their EHR data. Those were validated in a group meeting with other researchers, unfamiliar with Discovery, to ensure the themes reveal how Discovery was able to support the sensemaking and what improvements are necessary to support the patients’ sensemaking needs.

We noticed that there was saturation in the themes we observed—no key points were being brought up as we approached the end of the analysis. This was a signal that we obtained enough insights for concluding the study and that we can move toward reporting the results.

## Results

### Overview

The participants ranged from those that considered themselves healthy (6/14, 43%), through those who had episodes of frequent doctors’ visits for acute conditions (4/14, 29%), to those who had to manage ≥1 chronic diseases such as diabetes, high blood pressure, and asthma, among several others (8/14, 57%). The mean age of the participants was 33.43 (SD 10.39; range 20-53; median 30) years. Of the 14 participants, 6 (43%) were male and 8 (57%) were female. Some participants had very few medical records with 1 or 2 providers (2/14, 14%), others had an abundance of medical records scattered among multiple providers, from 5 or 6 up to a dozen (7/14, 50%), and the rest were somewhere in between (5/14, 36%). The participants that had rare encounters with their few providers generally found the patient portals useful and meeting their very basic needs. In contrast, those that had a lot of highly fragmented data across many providers found the experience very frustrating. Remembering how the portals work and manually pulling data together to prepare for clinical visits or just to understand their health status were reported to be very cognitively demanding and laborious. All but one participant reported being very comfortable with technology and using it daily. Most of the participants (11/14, 79%) had some experience in tracking their health data for which they used basic applications for running or steps count.

Our qualitative analysis resulted in the following themes: (1) *perceptions of Discovery: usefulness, usability, appearance, intuitiveness, and learning curve (RQ1);* (2) *sensemaking in the different views: data summary and overview, detection of time-oriented patterns, context while exploring, and comparison across providers (RQ2 and RQ3); and* (3) *sensemaking behaviors using Discovery: preference for single view, and preference for multiple views (RQ3).* In the remainder of the *Results* section, we report on these themes and provide 23 quotes from the participants labeled as P1 through P14.

### Perceptions of Discovery

#### Usefulness

Participants perceived Discovery as a very useful application with a multitude of use cases: preparing for and in clinical visits, consolidating medical records and comparing them across providers, tracking health events, quick access to health information, and raising awareness, reflection, and planning.

The participants liked how Discovery was able to pull the medical records from different providers in a single point of access. They generally liked the functionalities it has and the freedom of exploring their own medical records it brings:

I think now comparing it to MyChart, it’s so much more advanced. I can’t do all these things, pull up data and visualize it on a timeline, and check all the meds requested per year. All these, I can’t do on MyChart.

P14

However, several participants (3/14, 21%) found Discovery too complicated and technical for their needs. Despite that, all participants could immediately recognize how it can be used in their everyday lives. On the basis of what they reported, Discovery would be mostly used for raising awareness among the patients’ providers. Along these lines, participants wanted to use Discovery as an evidence-based platform for communicating with their providers in clinical visits or from home via messaging. Without exception, the participants said they would like to use Discovery to prepare for clinical visits and have the possibility to show the data they assembled to their physicians. With this, the participants expected to deliver their providers more complete insights about what they should know about them:

So, doing my due diligence [preparing and/or showing evidence] either before a visit or even while I’m on the ground at the visit, especially if they’re not within the same network. To be like, “Hey, I have had that and these were the results.” Would be really useful.

P2

Further, the participants reported they would use Discovery to keep track of their medical events over time such as vaccinations, laboratory tests, conditions, as well as trends of their vital signs and laboratory results. In addition, they saw Discovery used for consolidating their medical records and comparing the data across providers:

I think it really will be most useful in comparing data from different doctors, because I feel like within just the doctors I have, they have separate lab work.

P3

The participants envisioned they would also use Discovery to get insights in their health whenever they feel the need for it, do occasional reflections, and start planning on time and based on enough evidence. One important use case that stood out was using Discovery to keep track of what therapy worked or did not work for what condition and have that information readily available for comparison and sensemaking in case that condition happens again:

Let’s say I’ve got bronchitis again, and I’m trying to figure out what was prescribed to me the last time I got bronchitis, I can go to my records and see what I requested and I’ll say, “Oh, okay. I used acetaminophen.” So in the event that I seek a new provider that’s not UCLA or Pacific Alliance, I can tell them, “Listen, I know you can also see this in my records that I used acetaminophen.” But I would...Let’s say I didn’t want to use acetaminophen, let’s say it didn’t work for me.

P5

#### Usability

Discovery was able to support answering the sensemaking questions that, according to the participants, were reasonable and types of questions they would face in real life. However, owing to multiple views and customized UI components, users had to learn when to switch the views and master a relatively large set of new interactions in a short period. These sometimes led to suboptimal paths to answering the questions, spending time in deciding which view to use, or struggling with finding the answers (Multimedia Appendix 1 provides the detailed usability report).

Using the Summary view, all participants were able to easily and accurately answer the basic sensemaking questions (Multimedia Appendix 1) related to orienting themselves about the EHR data they were supposed to work with and the way the application operates using multiple views approach.

For the more focused sensemaking questions (Multimedia Appendix 1), the participants did take the desired approach and used the Catalog view. However, most of the times the Details Panel was the go-to feature for answering the questions and sometimes at the expense of the timeline widget, even for questions that had some temporality embedded in them. Participants attributed avoiding the timeline for multiple reasons: not wanting to learn a new widget when the one they already know (Details Panel) seems to be able to answer the question at hand, struggling with the intuitiveness of the timeline widget and being worried about the accuracy for the answers it provides (exact dates for example). Compared with the previous set of questions, we could observe that participants needed some trial and error to learn how the features in the Catalog view work and put more effort to find the answers. Most of the participants needed some help and assistance, but eventually were able to master the Catalog and provide correct answers to the questions, with rare exceptions when they did not or simply gave up.

For the last and most complex set of questions (Multimedia Appendix 1), we observed that participants overall still remained in the Catalog view. Although that was not the design intention, some correctly switched to the Compare view and the Timeline view depending on the questions. Those who were advised to try to switch to Compare, after spending some time in the Catalog, noticed efficiency gain for answering some of the questions and adjusted easily to the new layout. For the most part, participants were able to answer the questions correctly either with the Catalog or the Compare view. However, such positive insight was not present when they switched to the Timeline view for the last set of questions from this segment. Although they immediately felt that is actually the correct view and it seemed they had a mental model for how to tackle the questions, they faced a degree of disappointment when attempting to realize their strategy through the interface. This was mostly because of the cluttered interface and contradicting operations to what they experienced in the Catalog and the Compare. As a result, and taking into account the complexity of the questions tailored for the Timeline view, participants struggled the most there. For the most part, they needed step-by-step guidance to learn the interface and assistance in answering the questions. Even then, in some occasions, they were neither able to master this view nor were they able to find the correct answers.

#### Appearance

Some participants perceived the colors in the interface dull and somber and pointed out that they give a depressing mood, similar to the one in a hospital (4/14, 29%). To improve Discovery’s appearance, the participants requested more icons and vibrant colors that will make the UI look more friendly.

#### Intuitiveness

Participants found the Summary and Catalog views to be most intuitive and easy to use, followed by the Compare and then the Timeline view. However, they generally needed some help to figure out how and when to use the different views and the features within them. First, they registered some inconsistencies in how the same features behave in different views, which caused trial and error to discover the correct interaction principles for each of the views. Second, the participants noted that Discovery uses a number of customized, nonstandard UI controls and widgets that made them struggle to understand how they operate. Third, owing to the poor color selection in the UI, some of the controls were barely visible and indistinguishable from the surroundings, obscuring the affordances of the application:

Everything just blends in. It’s nothing that stands out because everything’s the same color. And the font is the same font. It’s not bigger or bolder. So I didn’t even notice [the timeline widget].

P1

Finally, few of the participants (3/14, 21%) pointed out that the language used to label the Cards and the details in them are too clinical and do not feel familiar to the user:

I think the reason why I was struggling earlier, when you kept telling how many times I got the flu shot in one given year, I think it’s because I know that the flu is called influenza, but I’m so used to seeing the word flu. And I feel like that’s what most people know it commonly as. So my question for you was is, I understand that this type of medical jargon has to be in the system because that’s how doctors understand the language. But do you think that for users who are more familiar with terms like flu instead of influenza, that you might include the word flu in parentheses for the users...And honestly, I think that both can coexist. It’s just a matter of how you’re going to display that language.

P5

#### Learning Curve

Although participants generally perceived Discovery as a very useful tool with powerful features that comprehensively support sensemaking, approximately half of them (6/14, 43%) commented that it might take a while until they get fully comfortable with it. This was primarily because of the multiple views they had to master and learn which view is most suitable for what types of questions:

And I do like the number of options that are given between the Catalog, Compare and Timeline. I feel like being a first-time user, probably didn’t have any awareness to make the most of it. I feel like if I was a patient using this for years on end, it is something I would learn to navigate pretty nicely and take advantage of.

P3

Few participants (3/14, 21%) were very skeptical that they would ever use Discovery to its highest potential as it simply offers more than they can handle, or even need:

There’s just too many different options. I really don’t feel like I need all these different visualizations [views]. I will choose only one mode or something. Just could be because I feel like even though it’s useful nobody...or I don’t know about nobody but I would never take the time to figure out how to use all the three different modes. I would just not spend time learning the software. I just want the information so I can get on with my life. I’m not going to be like, okay, let me spend an hour figuring out all the different modes and like that.

P12

In contrast, the rest of the participants (11/14, 79%) had a smooth transition to using Discovery for exploring and finding patterns in the EHR data, and for several of them (5/14, 36%), the multiple views posed no challenge:

It was easier for me. With the [multiple] views, it was easier for me to answer the questions, to be honest.

P8

Finally, more than half of the participants (8/14, 57%) emphasized the need of tutorial, help, and tool tips throughout the interface to familiarize the user with the way Discovery works.

### Sensemaking in Different Views

On the basis of the participants’ interaction with Discovery and their feedback, a number of important sensemaking activities surfaced: *data summary and overview, detection of time-oriented patterns, context while exploring, and comparison across providers.*

#### Data Summary and Overview

Participants were very pleased with the overview of their medical records the Summary view and Catalog view brought. Using the Summary view, they could easily see, on a high level, the different types of records they have and for what time span, as well as have a list of providers that have their records. Complementing this, the Catalog view provided a more granular and comprehensive overview of their data.

In contrast, although they appreciated the additional time dimension for the data overview the Timeline view introduced, most of the participants (9/14, 64%) felt overwhelmed by its appearance.

#### Detection of Time-Oriented Patterns

The Catalog and the Timeline views were primarily used for detecting time-oriented patterns in the data. The participants emphasized the importance of such capability as it enabled finding valuable insights in their medical events.

The participants especially liked the timeline widget in the Catalog view because it helped them get a better insight of when certain medical events happened, but also notice the time-oriented patterns of those events like prevalence, periodicity, and pre-post:

So, I also really enjoyed and appreciated the interactive aspect and how you can combine different data points yourself, to view. That was really helpful. And see the correlation between events and time.

P2

Further, the Timeline view added additional capability in detecting co-occurrence of medical events, bringing a full and robust way to detect time-oriented patterns and complementing the Catalog view:

I think, once you’re an experienced user, you’ll realize that you can answer questions a lot faster with Timeline view. I think Timeline might be the most efficient [for finding time-oriented patterns].

P13

However, the timeline widget showed flaws in some of its aspects. According to the participants, the color-coding of the dots in the timeline that represent the medical events was confusing and made it difficult to distinguish what is selected and what is not. Further, it was generally hard to interact with the Timeline view interface efficiently. Participants noticed that here, the timeline widget operates differently—the dots represent a particular day and they are clickable, whereas in the Catalog and Compare views, they are inactive. On top of that, they observed that the Details Panel in the Timeline view has browsing support characteristic, but it is a collection space for the records of interest in the Catalog and Compare view.

#### Context While Exploring

Participants were very pleased with the context Discovery provides during the data exploration in the sensemaking process. They valued the preservation of the broader context at all times in the Cards from the Catalog and pointed out it was an advantage over other applications.

All participants agreed that the Catalog view is their first option for exploring their data and should be the view Discovery is built around. They liked how the data are hierarchically organized and laid out for easy browsing:

When I went into the Catalog I liked the simplicity of being able to click on different record types. I liked that things were in categories, medications versus conditions versus claims and so on. I did like that there was a timeline and that I could select and deselect various categories. That is a useful search feature.

P4

Similarly, the participants expressed positive sentiment toward the Details Panel (12/14, 86%) that enabled preservation of a narrower context important for the sensemaking task at hand. They liked how the Details Panel represents a collection of relevant records, shows their details, and the visualization of their values over time when applicable. Another feature for capturing context in the Details Panel that couple participants (2/14, 14%) picked up and immediately respected were the free text notes for the records that could be used for reminders, additional explanations or missing details:

I like the idea that you can take notes. [examples follow]...it would be nice to also say: I was prescribed acetaminophen for bronchitis; it would be nice to say: This is why I'm prescribed this;...I took this, and I had a bad reaction. Don’t take this again.

P9

However, the Details Panel placement made it uncomfortable to use when the number of records in the panel grew—frequent resizing to reveal more of the Cards in the main view or more of the records in the panel made the experience unpleasant at times. In addition, some participants (4/14, 29%) requested more context during the exploration, such as more explanation about the labels and values in the individual records and linking between the records that are semantically related:

To me, if I’ve been diagnosed with a condition, I want to be able to click on it, see what medications were prescribed and also see doctor’s notes about it.

P10

Although the participants were generally very satisfied with the context management in Discovery, the way the Timeline view was laid out to show the broader temporal context of the data was perceived as off-putting:

The Timeline [view], yeah, it reminds me of like the little abacus that no one really uses anymore...Yeah, it’s not organized well, it’s muddled, like I said before.

P11

This impression was mostly related to the dedicated, separate timelines, for each of the record types. The problem with these record type timelines was that they were still present, albeit dimmed, even when the user had certain record types filtered out. This was contradicting the scoping principle from the other views where the filtered out records are never shown:

See now, when I click on benefits, it hides. It doesn't even take it away. So that’s even more visually annoying. Because it’s still there, but it’s just hidden, but not hidden very well...It should disappear. If you’re going to click it, you want it to disappear.

P10

One important suggestion that came from few participants (3/14, 21%) was to improve the browsing experience by allowing more context on demand for the date-dots in the Timeline view. The idea was to show details for the events (records) from that dot (day) on hover, in a box right next to it instead in the Details Panel, thus eliminating the need to constantly go between the main view and the Details Panel:

No, I think this view and that opening and closing the window like a blind, I think it’s really unnecessary. If you’re going to do a timeline, I would say you click on 2009, or you hover on 2009, a window comes up maybe with a summary of everything as far as all these categories you have from benefits to vital signs with individual dates.

P10

#### Comparison Across Providers

Participants mostly relied on the Catalog and Compare view to do comparison of the data across providers. They recognized the Compare view as a natural extension of the Catalog view that allowed them to compare the medical records across providers more efficiently:

And this is a little bit more detailed than the Catalog view, the Compare view, in my opinion. So if the question was more specific, like how many emergency room admissions and stuff have you had at Pacific Alliance or at UCLA Medical Center, this would be more detailed and more visually clear [in the Compare view].

P14

However, the participants identified some inefficiencies in the Compare view as well. First, there was no way to compare the medical records at record type level, so the participants had to visually infer the comparison by looking at the subtypes from the record type. Second, the sparklines that showed the distribution of medical events next to the Card lacked dates labeling, which made it difficult for the participants to interpret their meaning and how they correspond to the main timeline at the top.

### Sensemaking Behaviors Using Discovery

A smaller number of participants were satisfied with the multiple views approach for data exploration (5/14, 36%). However, the rest of them (9/14, 64%) found difficulties in determining which views to use for what questions and almost all (8/9, 89%) wished for a consolidated single view that preserves the functionalities from the others.

#### Preference for Single View

Participants liked the functionalities that Discovery provided. However, having multiple views for data exploration was found counterproductive for the most part. Although there were few that liked the multiple views approach (5/14, 36%), most of the participants (9/14, 64%) complained that it was difficult for them to keep in mind that there is more than one way to look at their data and even more challenging to decide which view to use for which question. This was even more frustrating and confusing to some participants (6/14, 43%) as it was apparent that there is some overlap in functionality between the different views:

I think that once you get in a certain mode, you want to stay in a certain mode. I know that was kind of the instance for me. I just wanted to stay the one that I knew. So it was a little bit difficult to change modes and be like, ‘Whoa, what is this?’ Or constantly go back to that mindset because if you were to go on a different mode, I feel like you’re completely changing my mindset. So you’re like, okay, which mindset am I at here? Which mindset am I at there? My way of thinking.

P6

Those participants who disliked the multiple views had the preference to stick with one view, master it, and try to answer the questions with that view, instead of learning how to use additional views that felt redundant to a certain extent:

So, for me, I mean, I’ll tell you right now, for me, if I learn how to use a part, my natural instinct would be, if I can get the information in a space I understand, I’m never moving.

P4

However, some of these (3/9, 33%) participants did acknowledge that given an extended period to use Discovery, the mapping between a question and a view most suited to answer it would become less demanding. However, few participants (3/14, 21%) questioned if they would actually be motivated to use Discovery that heavily to reach such a point of mastery—they wanted to have very simple and basic functionalities that they can use sporadically.

Given all this, the general sentiment of the vast majority of participants (11/14, 79%) was that we should preserve the features that the views offer, but collapse the multiple views into a single one for simplicity and to avoid confusion:

I think I would prefer maybe just have, yeah, one view and then maybe have some of those other features integrated in that same view because of the overlap [of the features in the different views].

P7

#### Preference for Multiple Views

In contrast, there was a different, much smaller camp of participants who liked the multiple views and did not find it difficult to learn how to use them (5/14, 36%). Some also saw value in having the option to rely on a view that fits one’s analytics skill set and mental model of the data (2/5, 40%). For example, the Catalog view for those who prefer more tabular look of the data versus the Timeline view for those who are primarily interested in the time orientation of the data:

Ideally, I think it’s great to be able to see your records in a variety of ways.

P10

Although exerting some effort to find the most appropriate view for the given question, these participants did find a lot of strength in the multiple views approach. They were very confident they would be able to easily master how to use the multiple views to the maximum of their potential given more time:

I feel this being my first time seeing it, I, I don’t know, got stuck in the tab I currently was on, but I think that once I switched—just having these other views did make it easier to answer questions more quickly and more efficiently.

P3

## Discussion

### Principal Findings

To our knowledge, this is a first study that digs deep into how patients go about making sense of EHR data from multiple providers, what features they desire to support that process, and what the use cases might be.

In this evaluation study, the participants found most of the features in Discovery useful and easily identified use cases applicable to their everyday lives such as clinical visits, raising awareness, reflection, and planning. The participants agreed that the functionalities Discovery offers are very useful and important for supporting comprehensive sensemaking: summary and quick overview of the data, finding prevalence, periodicity, co-occurrence, and pre-post of medical events, as well as comparing medical record types and subtypes across providers, anchored to a timeline. Although they saw value in each of the views, participants strongly favored the Catalog over the others and wished for a consolidated single view, centered on its layout, that preserves the functionalities the rest of the views offer.

However, although deemed exceptionally useful, the application was often perceived as complex and offering too much. The multiple views for data exploration likely contributed to this perception. In addition, the intuitiveness and the learning curve for the application did not meet the expectations, owing to the experience needed to switch between views depending on the information need, inconsistencies of interactions in different views, and customized, nonstandard UI components throughout the application. Finally, the color schema and the language in the interface set a clinical tone that participants wanted to be replaced with more user friendly labelings, vibrant colors, and iconography.

### Interpretation of the Results and Contributions

The perceptions and preferences for the features should be perceived form 2 points of view: time spent with the application and fidelity of the medical records used for the study. First, there is the limited time that was made available for getting familiar with the application. This might have caused the participants to optimize their use of the application by what features look most familiar and require less experimentation and learning. In addition, aside from the help that the evaluator provided when participants struggled, there were no tooltips, reminders or nudges about how should the interface be used for completing important tasks, which are typical practices in widely adopted systems like email and social media. Given more time and pervasive help, it is conceivable that participants might have developed different perceptions and attitudes toward the features in the application. This is especially true for powerful widgets, like the timeline, that take some time to get familiarized with—often their full benefit surfaces only if the necessary learning time is invested. If that does not happen, they look less useful and may cause frustration and confusion. Similar applies to developing a mental model of how the application is intended to operate on a higher level. For example, the multiple views approach might have been less confusing if the participants had substantial time to spend interacting with the application based on their personal questions for their own data.

This brings us to the second point for interpreting the results, which is related to the fidelity of the data in the study. The participants engaged with synthetic data for a fictitious patient. This unfamiliar data set could have additionally burdened their sensemaking since they were likely not acquainted with the clinical terms present in the data the same way they might be for their own. We could expect that if participants worked with their personal data and questions that trouble them, the motivation and the strategy to engage in sensemaking could have been different. Therefore, the utility of the features and specialized views might have changed. Finally, we only presented participants structured clinical data without the notes from the physicians. It is well known that clinical notes glue the different data types together and add them meaning. For these reasons, we still do not know how the availability of clinical notes might influence the sensemaking around structured clinical information and how will the availability of clinical notes change the sensemaking needs.

Being respectful to the points for interpreting the reliability of the results, this study still produced valuable contributions. Our work is different from other studies on single provider patient portals that focus on features use and list patients’ desired features for interacting with their data [6,20]—we found granular insights in how patients go about making sense of their EHR data from multiple providers. Our findings showed that the time represents an anchoring component in the sensemaking process. This principle is neglected in most of the current solutions that heavily rely on lists of various record types, without being linked to a timeline for finding time-oriented patterns in the data [22-24]. Going deeper, participants were very interested in finding co-occurrence and periodicity of medical events, as well as learning what happened before or after a particular medical event. Current solutions usually can surface the periodicity for a single variable by plotting longitudinal record types such as vital signs and laboratory results [22,24], but fail short to support the other time related needs robustly.

Participants also wanted to browse the data quickly and efficiently, but at the same time required variable context depending on the stage of the sensemaking. This context included the records being laid out at all times in the user view, having more information about an individual record, understandable explanations about values in the records, as well as identifying other relevant records to the one in focus. Although existing applications provide explanations for medical concepts and abnormal values [24,25], they lack the richer context that surfaced from the user needs in this study. Finally, comparison of the data by provider for a specific record type or its subtype, or a subset of them, was in demand, however such capability is not fully addressed in existing solutions.

This study showed that Discovery is able to, at least partially, support most of the previously listed sensemaking needs that are not addressed in other solutions. Detecting prevalence, periodicity, co-occurrence of events, and pre-post analysis could be achieved with the Catalog and the Timeline view, deeper and persistent context can be achieved with the collected records of interest in the Details Panel in combination with the records’ notes, and efficient comparison across providers could be performed in the Compare view. However, the study indicated much room for improvement to meet the patients’ sensemaking needs even more closely.

Although the study produced numerous contributions around patients’ needs for making sense of their EHR data, we never deeply explored how Discovery might affect the patient-provider communication and if the sensemaking support features in Discovery could benefit physicians as well. It would be interesting to explore how Discovery can play a role as a common ground setting and evidence providing instrument in clinical visits and in asynchronous messaging between the patient and the physician. To corroborate this interest, there is evidence from this study that participants were strongly motivated to use Discovery in the communication with their physicians. This is an important topic that deserves serious attention in a separate endeavor. Such dedicated approach is necessary, as Discovery needs to move in the direction of improving and supporting existing workflows and not aggressively interrupting the current practices and causing additional confusion in the communication.

For these reasons, our design implications will focus more narrowly on improving the sensemaking for the patient, leaving the support for communication with the physicians and improved shared decision-making for later efforts.

### Design Implications

#### Design Principles

On the basis of the experience of this study, we want to offer 3 general guidelines for designing patient-facing sensemaking tools that handle high user and data variability. The first guideline is to address the core of the user needs, those most important and prevalent ones that target the widest population, in a very simple and intuitive fashion. The second guideline is introduction of advanced features in a layered approach starting from more prevalent and important ones to very specific and sporadic. The third and final guideline is that at the very first new user interaction the interface should give the impression that it can bring considerable value extremely simply and intuitively. The user should then be able to easily start expanding the interface with new features or collapse those, as needed, but never feel that unnecessary complexity is thrown at them without a choice to avoid it.

We want to point out that achieving design goals set forth with these guidelines will require a very tight design-implementation-evaluation loop in which it will be essential to carefully solicit and analyze the users feedback.

The upcoming design implications are following these recommendations and possibly contribute to improved patient-facing sensemaking support applications.

#### Making Discovery More Intimate—Personalization and Familiar Interactions

As Discovery is supposed to be the window to the patient’s medical data, we propose that the experience of using the application has to be highly personal and intimate. It has to resemble a sense of trust and pleasurable environment, which yields that making sense of EHR data should not be a dreaded experience that takes place closely before or after interfacing with the health care system [14,32]. Instead, it should be a comfortable process of continuous learning from these data to support health improvements.

First, instead of dull and somber colors that trigger association to hospitals, we need to use more lively colors that create associations with nature, life, and hope.

Second, instead of using clinical terminology, we need to rely on language that the everyday patient can easily understand. For example, the user can work with Discovery using the consumer language, but should also be able to see the precise clinical terminology on demand. This is important for merging and comparing data from different providers, and more importantly in clinical visits when medical professionals have to look at the data as well. To further observe the application as an extension of self, rather than an alien agent, we could offer the user to create labels for the EHR data (medications: blood pressure pills, laboratory tests: fat in blood, immunizations: flu shot, etc), as it is known that people build their own vocabulary around their health that makes it easier to think and talk about it [7]. Similar to the previous case, mappings between user generated labels and the clinical ones will also exist, for the same reasons. In addition to a user-friendly language for the medical records and labels throughout the interface, we should provide explanations for those clinical terms that cannot be easily translated for the consumers or carry meaning that is not assumed common knowledge.

Third, the interface of Discovery should use standard UI components and interactions that patients are familiar with from popular applications such as email, photo albums, or social media. This will potentially increase the intuitiveness of the application and flatten the learning curve. This will give the impression to the user that they are interacting with Discovery as a confidant that knows and understands them from the get go, instead of a stranger with whom they struggle to communicate.

Finally, we need to ensure that there is pervasive support for the users to learn how to use the interface. Short video tutorials in a specially dedicated YouTube channel are one possible approach. In addition to this, help should be available in the application itself and be context-dependent. In addition to being able to look for explanations as needed, tooltips can be offered as the user explores new interactions. This will be especially relevant for highly useful features that might have a limit to their intuitiveness as they take new approaches to solving nonprevalent, but important, domain-specific problems.

#### Consolidation of Multiple Views Into One, but Preserving the Functionalities

Respecting the feedback from this study, efforts should be made to provide a single view for data exploration that embodies a variety of functionalities: data comparison across providers and time-oriented pattern detection for medical events, with capabilities of providing context on demand for the individual medical records as the sensemaking unfolds.

Fortunately, the modeling in the presentation layer of Discovery enables us to quickly pivot the design to achieve a single view data exploration. Anchoring this view in the Catalog layout, we could simply inject the Compare view sparklines to the right of the Cards on-demand, eliminating the need for separate comparison view. To support robust time-oriented pattern detection and eliminate clutter in the UI, we can enable on-demand highlighting of different individual records (or record subtypes) in the timeline widget using markers with different colors and having them stacked on top of each other if they occurred on the same day. This capability would introduce the detection of co-occurrence of events that was lacking in the Catalog and simply allow us to eliminate the Timeline view. The only step that is left for features preservation in a single view is adding interactivity to the timeline widget to highlight the Cards that contain records from a given date, once the date-dot in the timeline is clicked. For additional improvement based on the participants feedback, we can add labels to the sparklines and details on hover for the date-dots.

#### Efficient Exploration with Context While Browsing

More context needs to be provided on demand as the browsing takes place. First, explanations are needed in addition to the values that are presented, primarily for vital signs and laboratory results. Second, Discovery is only showing a very limited set of attributes for a given record. Future design should consider uncovering details on demand as the patient makes sense of their data. Finally, in the current representation, Discovery generally assumes independency between individual records. There are 2 exceptions to this notion: for longitudinal data such as vital signs and laboratory results (for which it plots the time series in the Details Panel) and co-occurrence of events that took place on the same day (for which it has a date-dot in the Timeline view that bundles them together). However, the dependencies between the medical records can be much richer than that: records from the same encounter or records related to a certain condition, records within a time frame around an event, and records to also consider if looking at a specific record with a certain value, just to name a few. Therefore, there is an opportunity for deriving more context on demand or when the system thinks it is necessary [6].

In Discovery, this could be achieved by allowing layered expansion of the record’s attributes in the Details Panel based on the user needs, adding suggestions for other records next to the record’s details, and by enabling filtering records related to a selected condition, records that happened in the same encounter or within a given time frame around an event.

### Limitations

This study has several limitations. First, we conducted a remote study, which possibly attracted individuals comfortable with applications for web meetings. Moreover, we used a digital platform for recruitment, Craigslist, which could have additionally biased the sample toward a tech savvy population.

Second, the selection of the study format and recruitment platform might have contributed to a sample size biased toward the younger population, while older adults were not included. However, we believe that the sample in our study was fairly representative of what potential users might be. In contrast, we believe that the design of sensemaking support applications for older adults requires special attention and a separate approach. Therefore, it will be interesting to evaluate Discovery exclusively with that population and see what the feedback will look like and what design implications can be drawn from that.

Third, the evaluation was conducted on synthetic data from a fictitious patient. This set-up might have contributed to decreased motivation to engage in sensemaking using Discovery and learning how to use its features or omission of potential real-life use cases. For these reasons, in future evaluations, we are strongly committing to use the EHR data of the study participants.

Fourth, the participants did not go through any detailed tutorial for using Discovery and had only 30 minutes to interact with it and learn how to use it as they were progressing through the study. These relatively difficult circumstances might have caused some of the negative feedback on the multiple views intended for sensemaking, the learning curve, and the intuitiveness of the tool. Future studies should complement this one where the participants not only engage with their own data, but do that over an extended period, while their interactions with Discovery are being logged for analysis.

Finally, despite the aforementioned limitations, we are convinced that this study produced valuable pioneering insights about the design of patient-facing sensemaking tools for EHR data from multiple providers.

### Conclusions

On the basis of this study, patient-facing sensemaking support tools such as Discovery should support high variability of users and data with a very intuitive and easy to use interface. This interface should support completing the most important and prevalent tasks in its simplest form, but at the same time be easily expandable to more advanced features as the needs get more complex and the users get more comfortable with the tool. The user should be able to detect time-oriented patterns of medical events in their data while getting enough context on demand for making sense of the individual medical records. All that should be achieved in a single exploration view that feels familiar, warm, and positive, and has enough plasticity to adjust to the information needs of the user as the exploration unfolds.

In addition, adoption of tools such as Discovery is equally important as the benefits they have the potential to bring. Pervasive and easily accessible help in various formats and understandable, useful examples of use should be available to wide populations of patients. This should assist in learning and accommodating to the new ways of interacting with and using their medical records.

Finally, establishing methods for efficient design, development, and evaluation of sensemaking support tools will be essential for their success. Making efforts related to securing larger groups of study subjects, various cohorts, and mechanisms for longitudinal evaluation on patients’ own EHR data will be the key for the validity of these studies.

In the future, patient-centric sensemaking support tools should explore how to include the physicians in the process and improve the shared decision-making. These tools should be adjusted to include clinical notes and incorporate those as an important component for the patients’ sensemaking. Further, we should explore how to introduce patient empowering tools such as Discovery in a way that does not materially interrupt the existing clinical workflows. Future tools should leverage the design principles and implications from this study, but their designs should also take a step further in carefully tailoring ways how to incorporate them in clinical practices such as clinical visits, remote consultations, and messaging.

Paradigm shifts toward patient empowerment and partnership with the physicians should happen gently. Features in tools that support this should be gradually introduced, respecting the preferences of wide patient populations and existing constrains in clinical workflows within the health care system.

## Appendix

Multimedia Appendix 1 Detailed usability report.

## Abbreviations

EHR: electronic health record
FHIR: Fast Health Care Interoperability Resources
RQ: research question
SMART: Substitutable Medical Applications and Reusable Technologies
UI: user interface

## Data Availability

The data sets generated during or analyzed during this study are not publicly available owing to the privacy and safety commitments in the institutional review board protocol and the consent form that the study participants signed. The researchers stated that only the research team may have access to the study interviews and only the results would be shared.
