# Supplementary material for: New Ways for Patients to Make Sense of Their Electronic Health Record Data Using the Discovery Web Application: Think-Aloud Evaluation Study
Source: JMIR Form Res. 2023 Apr 3;7:e41346. doi: 10.2196/41346 (PMC10131650; doi:10.2196/41346)
Supplement: Multimedia Appendix 1 [file formative_v7i1e41346_app1.docx]

Table 1. Difficulty and approach for accomplishing the sensemaking tasks.

| **Sensemaking tasks for evaluating Discovery** | |
| --- | --- |
| 1. Basic questions (overview of data, functioning of Discovery) | |
| For what time span is there data available for you (Adela)? | Easy to find the answer for everyone using the Summary View. |
| From how many providers is there data about you? What are the provider names? | Easy to find the answer for everyone using the Summary View. |
| What is the total number of records available for this patient? | Easy to find the answer for everyone using the Summary View. |
| How many different views are there to look at the data? What does each do? | Using the Summary View, a little struggle for some participants to conceptualize the idea of multiple views and figure out what each of them is doing. |
| 1. Focused questions (lookup, prevalence, frequency, details) | |
| How many times did you have flu shot before? Did you have it last year? Did you ever have it more than once in a single year? | Everybody used the Catalog View. Almost all relied on the Details Panel, after making a few interactions to learn how the Cards work. After opening the Card for the flu, they scrolled up and down through the Details Panel to find the answers. Except for the excessive time and energy applied to the scrolling, almost everyone got the right answers. |
| How many different immunizations did you have? What about in the last 5 years? First 5 years? | Everybody used the Catalog View. Here, still the majority of participants used the Details Panel to find the answers after selecting all Immunization Cards and scrolling up and down, and counting. However, few went for the Timeline as their first option. Those participants struggled to figure out how the widget operates: narrowing down to a 5 year window was not apparent and easy. Eventually, with some explanations and nudging, everybody was able to find the answer whether using the Details Panel or the Timeline widget. |
| How many of the different immunizations came from the UCLA Medical Center? | Participants had different approaches for this – using the Providers filters first or simply relying on the Details Panel without filtering: scrolling through the Immunizations and counting the ones that came from the designated provider. Regardless of the strategy, everybody found the answer successfully. |
| How many times did you have ***HPV, quadrivalent*** immunization? | This was easy to figure out from the corresponding Immunization Card itself, or by counting instances in the Details Panel. Most of the participants did it relying on the Card. |
| What is the status of each ***HPV, quadrivalent*** immunization? | Everybody relied on the Details Panel and was successful to find the answer. |
| When was the first time you had a ***meningococcal MCV4P*** immunization? | After clicking on the corresponding Card, most of the participants relied on the Details Panel, but some used the Timeline. Relying on the Details Panel gave them more confidence to give precise answer to the question. Because the date-dots in the Timeline widget were not interactive to show mor detail, the date for the medical events could only be approximated based on the labels in the visualization. All participants gave satisfactory answers. |
| 1. Complex questions (comparison across providers, cooccurrence, pre-post) | |
| Can you compare the number of encounters you had with both of your providers? | Most of the participants stayed in the Catalog View. They had different approaches for this – using the Providers filters first to inspect one Provider at a time, or simply relying on the Details Panel without filtering, after selecting all Encounters Cards. However, a few participants intuitively switched to the Compare View. Those who switched found the answer much more efficiently by relying on the sparklines to the right of the Cards. Regardless of the strategy, everybody found the answer successfully. |
| Were there years when you encountered any provider very frequently? What period of time was that? | Of those who initially stayed in the Catalog, some decided to explore the Compare view or were simply nudged to do that. Similarly, while they were able to find the answers in the Catalog view, those who switched to the Compare did it more efficiently. Interestingly, while they were able to quickly identify the density of Encounters from the sparklines, it was hard to map that to an actual time frame due to the absence of any labeling in the sparklines. Rather, they had to make visual mapping from the sparklines to the Timeline widget, which felt like loss of accuracy in the answers. All participants gave satisfactory answers. |
| When did most of your procedures take place? | Staying in the Compare View and relying on the sparklines didn’t bring any significant benefit for answering this question as compared to the Catalog. The reason for this was the absence of sparklines for the entire Record Type – participants had to visually combine the sparklines for different Procedures. While this was faster than clicking each individual Card (like in the Catalog), it was also less accurate. However, all participants were able to provide satisfactory answers. |
| Were there any medications requested when you were diagnosed with ***acute bronchitis (disorde****r)?* What about requests within a year of that event? | Participants mostly stayed in the Catalog, but some intuitively switched to the Timeline View. Those who stayed in the Catalog went through numerous interactions to find the correct answer: starting with identifying the dates when they had bronchitis, then trying to find medications for that date. Some of them were unsuccessful or gave up. Those who switched to the Timeline felt like they have strategy how to tackle the problem, but faced challenges in executing their plan with the available user interface components. The presence of individual Timelines for all Record Types overwhelmed the participants, and it was not straightforward where to look for bronchitis or identify which of the Conditions was acute bronchitis. The vertical alignment that captured cooccurrence was not always intuitive for the participants. As they received step-by-step explanations how the interface works and guidelines how to answer the questions, they started feeling the power of the Timeline View more and more. Even then, in some occasions, they were not able to master this view nor were they able to find the correct answers. Consequently, they emphasized that more effort needs to be made to make the interface more intuitive and appealing, with addition of some functionalities like providing details about the date-dots on hover. |
